# Generation and Characterisation of a *Pax8*-CreER^T2^ Transgenic Line and a *Slc22a6*-CreER^T2^ Knock-In Line for Inducible and Specific Genetic Manipulation of Renal Tubular Epithelial Cells

**DOI:** 10.1371/journal.pone.0148055

**Published:** 2016-02-11

**Authors:** Judit Espana-Agusti, Xiangang Zou, Kim Wong, Beiyuan Fu, Fengtang Yang, David A. Tuveson, David J. Adams, Athena Matakidou

**Affiliations:** 1 Department of Oncology, University of Cambridge, CRUK Cambridge institute, Cambridge, United Kingdom; 2 Transgenics Facility, University of Cambridge, CRUK Cambridge institute, Cambridge, United Kingdom; 3 Experimental Cancer Genetics, Wellcome Trust Sanger Institute, Wellcome Genome Campus, Hinxton, Cambridge, United Kingdom; 4 Cytogenetics Facility, Wellcome Trust Sanger Institute, Wellcome Genome Campus, Hinxton, Cambridge, United Kingdom; The Roslin Institute, UNITED KINGDOM

## Abstract

Genetically relevant mouse models need to recapitulate the hallmarks of human disease by permitting spatiotemporal gene targeting. This is especially important for replicating the biology of complex diseases like cancer, where genetic events occur in a sporadic fashion within developed somatic tissues. Though a number of renal tubule targeting mouse lines have been developed their utility for the study of renal disease is limited by lack of inducibility and specificity. In this study we describe the generation and characterisation of two novel mouse lines directing CreER^T2^ expression to renal tubular epithelia. The *Pax8*-CreER^T2^ transgenic line uses the mouse *Pax8* promoter to direct expression of CreER^T2^ to all renal tubular compartments (proximal and distal tubules as well as collecting ducts) whilst the *Slc22a6*-CreER^T2^ knock-in line utilises the endogenous mouse *Slc22a6* locus to specifically target the epithelium of proximal renal tubules. Both lines show high organ and tissue specificity with no extrarenal activity detected. To establish the utility of these lines for the study of renal cancer biology, *Pax8*-CreER^T2^ and *Slc22a6*-CreER^T2^ mice were crossed to conditional *Vhl* knockout mice to induce long-term renal tubule specific *Vhl* deletion. These models exhibited renal specific activation of the hypoxia inducible factor pathway (a VHL target). Our results establish *Pax8*-CreER^T2^ and *Slc22a6*-CreER^T2^ mice as valuable tools for the investigation and modelling of complex renal biology and disease.

## Introduction

Genetically engineered mouse models (GEMM) have been instrumental in understanding the basic principles of renal biology and disease. Relevant mouse models need to recapitulate the hallmarks of human disease by permitting gene alteration to occur specifically within relevant cell types, and appropriate stages of development. This is especially important for replicating the biology of complex diseases like cancer, where genetic events occur in a sporadic fashion within developed somatic tissues. Though a number of renal tubule targeting mouse lines have been developed, their utility in the study of renal cancer biology is limited by lack of inducibility (*Ksp*-cadherin Cre [1], homeobox B7 Cre [2], Aquaporin 2 Cre [3], γ-glutamyl transpeptidase Cre [4], phosphoenolpyruvate carboxykinase Cre [5] and Six homeobox 2 Cre [6]) and/or specificity (phosphoenolpyruvate carboxykinase Cre [5], and Paired box 8 rtTA [7]). To address this deficiency in the field we set out to generate novel inducible mouse lines allowing investigation of RCC biology by specifically directing genetic modifications in either the proximal tubule (the presumed cell of origin [8]), or throughout all renal tubular epithelial compartments.

Conditional gene expression systems provide an excellent way of controlling gene expression and circumventing any developmental defect as well as early lethality associated with conventional gene targeting. These models rely on the use of site-specific recombinases to control the spatiotemporal mutation of the genome. The most common conditional approach uses the bacteriophage Cre-Lox system, in which Cre recombinase recognises a pair of inverted repeat DNA elements, or LoxP sites, and catalyses recombination resulting in deletion or inversion of the intervening sequence [9]. Further temporal control can be achieved with an inducible Cre-recombinase, CreER^T2^, a fusion protein of the Cre-recombinase coding sequence and a mutant form of the ligand-binding domain of the oestrogen receptor (ER^T2^) [10]. Administration of tamoxifen forces the dissociation of the ER^T2^-bound heat shock protein 90 (HSP90), allowing CreER^T2^ to translocate into the nucleus and induce recombination.

Here we adapted the CreER^T2^ system for specific use in renal tubular epithelia. We describe the development and characterisation of two genetically engineered mouse lines that allow for spatial and temporal targeting of conditional mutations in either all tubular epithelial compartments (*Pax8*-CreER^T2^ transgenic mice) or specifically in proximal tubular epithelial cells (*Slc22a6*-CreER^T2^ knock-in mice). We further establish the utility of these lines for the study of renal cancer biology (a malignancy of the renal tubular epithelium) by generating models of renal specific deletion of the von Hippel-Lindau (*VHL*) gene, the most common genetic change found in human renal cancer [11–13].

## Materials and Methods

### Ethics Statement

All experimental procedures were carried out in accordance to Home Office UK regulations and the Animals (Scientific Procedures) Act 1986 (licence no. PPL 80/2465 and 80/2552). All experimental protocols were approved by the Animal Welfare and Ethical Review Body (AWERB) of the University of Cambridge CRUK Cambridge Institute. At the end of the study, mice were euthanized by cervical dislocation, in accordance with stated Home Office UK regulations.

### Mice

We used the following mice: C57BL/6J and CD1 from Charles River laboratories, B6.129S4(C)-*Vhl*^*tm1Jae*^/J (Jax: 012933), FLPeR [14] (Jax: 003946) and Rosa26R [15] (Jax: 003474) reporter mice from Jackson Laboratories.

### Construction of vector p*Pax8*-CreER^T2^

We used the p*Pax8*-rtTA plasmid [7] (kindly donated by Professor Robert Koesters, DKFZ, Heidelberg, Germany) and replaced a 0.9 kb AscI/HpaI restricted fragment containing the rtTA sequence with the CreER^T2^-pA (polyadenylation signal) sequence contained within a 2.2 kb AscI/HpaI fragment of the pCreER^T2^-pA plasmid [10] (kindly donated by Professor David Tuveson, Cancer Research UK Cambridge Institute, UK).

### Generation of *Pax8*-CreER^T2^ transgenic mice

For microinjection, p*Pax8*-CreER^T2^ was digested with PspXI/NotI and the *Pax8*-CreER^T2^ cassette (8.4 kb) was isolated via gel electrophoresis and gel extraction (Qiagen). The linear DNA fragment was then microinjected into fertilised C57BL/6J embryos using standard procedures. The *Pax8*-CreER^T2^ founder lines were bred with C57BL/6J wild type mice to determine germline transmission.

### Fluorescence in situ hybridisation (FISH)

We hybridised metaphase spreads from splenocytes derived from *Pax8*-CreER^T2^ transgenic mice with the *Pax8*-CreER^T2^ probe using standard protocols. The *Pax8*-CreER^T2^ probe was generated by incorporation with digoxigenin-11-dUTP (Roche) via PCR and detected with monoclonal mouse anti-digoxin (Sigma-Aldrich) and Texas red conjugated goat anti-mouse IgG (Molecular Probes). Images were captured using the SmartCapture software (Digital Scientific, UK) and metaphases were karyotyped using the SmartType Karyotyper software (Digital Scientific).

### DNA sequencing

Sheared genomic DNA isolated from the liver of a *Pax8*-CreER^T2^ transgenic mouse (1 μg) was subjected to Illumina paired-end DNA library preparation and PCR-amplified for six cycles. Amplified libraries were sequenced using the HiSeq platform (Illumina) according to the manufacturer’s protocol. Paired-end, 75 base reads were generated to an average of 3.5 fold coverage across the genome. In order to identify the insertion site of the *Pax8*-CreER^T2^ transgene, sequencing reads were aligned with BWA version 0.5.10 [http://www.ncbi.nlm.nih.gov/pubmed/19451168] to a modified C57BL/6J reference genome (GRCm38/mm10) with the chromosome 2 *Pax8* gene sequence removed and the *Pax8*-CreER^T2^ transgene included as a separate contig. Read alignments to the *Pax8*-CreER^T2^ transgene were visualized using the Intergrative Genomics Viewer [16] to determine the chromosome and position where the read mates were clustered, indicating the site of transgene insertion. Sequencing data can be downloaded from the European Nucleotide Archive website under sample accession number ERS400266.

### Construction of vector p*Slc22a6*-CreER^T2^-FHF

We used recombineering [17, 18] to retrieve a 10kb fragment from a mouse C57BL/6 background Bacterial Artificial Chromosome (BAC; RP23-457i11) containing the *Slc22a6* locus. Briefly, the plasmid pSC101-BAD-gbaA^tet^ was electroporated into *E*. *coli* containing the RP23-457i11 BAC to provide the recombinase. We activated the recombineering machinery and introduced by electroporation a pBS vector containing two previously cloned 80 bp retrieving arms (a 3’ arm and a 5’ arm) homologous to the ends of our chosen 10 kb *Slc22a6* BAC region. This induced retrieval of the *Slc22a6* locus by homologous recombination between the BAC and the 3’ and 5’ arms of the pBS vector. The resulting vector (pBS-*Slc22a6*) consisted of the *Slc22a6* exons 1 to 6 (4 kb), 4.3kb upstream genomic sequence and 1.3kb of intron 6. We PCR amplified 487 bp of the pBS-*Slc22a6* plasmid including the gene start codon and 400bp of upstream sequences using the 5’ primer 5’-CATCGGTACCCATCCTCCCTTGCCCTTCATT-3’ and the 3’ primer 5’-TCGACCGGTAATGCAGGCAAATTTTGGTGTACGGTCAGTAAATTGGACATGGGGCTGGGCCAGGCTGAGTGGTCAAT-3’. After cutting the PCR-fragment with KpnI/AgeI it was ligated into the KpnI/AgeI site of the pCreER^T2^-pA plasmid (p5’UTR-CreER^T2^) replacing the start codon of the *Slc22a6* gene with the start codon of the CreER^T2^ cassette. A synthesised oligonucleotide (Sigma, UK) containing unique HpaI and PacI digestion sites (5’-GGTCACCGTTAACGCACAATGGCACAGAGGCCATCACAATGGCACAGAGGCCATTAATTAAGGTCACC-3’) was ligated into the BstEII site of pBS-*Slc22a6*. The MfeI/PacI sites were used for the ligation of a 2.6kb MfeI/PacI fragment of p5’UTR-CreER^T2^ re-instating the endogenous 5’ sequence of the mouse *Slc22a6* locus and replacing the gene start codon with the CreER^T2^ open reading frame (pBS-*Slc22a6*-CreER^T2^). Finally an FRT-PGK-Hygromycin-pA-FRT selection cassette (kindly donated by Professor David Tuveson, Cancer Research UK Cambridge Institute, UK) was ligated into the HpaI/PacI sites of pBS-*Slc22a6*-CreER^T2^, 3’ of the CreER^T2^ sequence and 5’ of the remaining endogenous *Slc22a6* exon 1 to generate p*Slc22a6*-CreER^T2^-FHF. The identity and integrity of the final plasmid was confirmed by Sanger sequencing.

### Screening of electroporated ES cells

C57BL/6J embryonic stem cells (CRIB6.1, an ES line derived in house) were electroporated with the p*Slc22a6*-CreER^T2^-FHF targeting construct. The ES cell colonies were then screened for correct locus insertion by PCR amplification of: (1) a 4.6 kb DNA fragment using a forward primer located upstream of the 5’ homology arm (5’-GCAGTGGTCCATTTAGCACA-3’) and a reverse primer located in the CreER^T2^ cassette (5’-CAGGTTCTTGCGAACCTCAT-3’) and (2) a 5.2kb DNA fragment using a forward primer located in the hygromycin selection cassette (5’- AGCTTGTCGACGAAGTTCCTA-3’) and a reverse primer located within exon 7 of the *Slc22a6* gene (5’- GCCGAAAATCACCTGGATAA-3’). The PCR conditions were set as follows: 1 cycle of 1 min 95°C, 30 cycles of (15 sec 95°C, 30 sec 58°C, 5 min 68°C), 1 cycle of 7 min 68°C.

ES cell colonies that tested positive by PCR for correct insertion of the targeting vector into the *Slc22a6* locus were grown further and DNA was extracted using standard protocols. Purified DNA was digested with SpeI/EcoRV (for 5’ probe detection), HindIII (3’ probe) or SpeI (internal probe) and used for Southern blotting. The 595bp 5’ external probe hybridised upstream of the 5’ homology arm of the targeted locus and was amplified by PCR from wild type C57BL/6J DNA using the following primers: forward 5’- AGCAGTTTTGGAAAGGCTTC-3’ and reverse 5’- CCCTTGATGATCTTGTGGTTC-3’. The 590bp 3’ external probe hybridised downstream of the 3’ homology arm of the targeted locus and was amplified by PCR from wild type C57BL/6J DNA using the following primers: forward 5’- AAGGCTGTCTGGCTTCCTCT-3’ and reverse 5’- GACCTCTCAGGCCTTTGACA -3’. Finally the 579bp internal probe hybridised to the hygromycin selection cassette of the targeting vector and was PCR amplified from it using the following primers: 5’- GATGTTGGCGACCTCGTATT-3’ and reverse 5’- GATGTAGGAGGGCGTGGATA-3’. The probes were labelled using NEBlot kit (NEB) and Southern blotting was performed using standard protocols.

### Generation of *Slc22a6*-CreER^T2^ knock-in mice

Correctly targeted ES cells were karyotyped and injected into 8-cell embryos of CD1 mice, which were then implanted into pseudopregnant CD1 females (Cancer Research UK Cambridge Institute Transgenic core). Chimeric founders were mated with wild-type C57BL/6J mice. Genotyped progeny that were positive for germline transmission were bred, and first generation offspring that inherited the targeted allele with hygromycin were subsequently mated with C57BL/6J mice. The hygromycin selection cassette was removed using FLPeR mice [14]. The FLPeR was bred out of the mouse line after hygromycin cassette elimination.

### Genotyping

Mouse genotypes from ear biopsies were determined using real time PCR with specific probes designed for each gene (Transnetyx, Cordova, TN). Additional genotyping using PCR was undertaken for the presence of Cre-recombinase (forward primer 5’- GCACTGATTTCGACCAGGTT-3’, reverse primer 5’- GCTAACCAGCGTTTTCGTTC-3’, 200bp product) and hygromycin sequences (forward primer 5’-GATGTTGGCGACCTCGTATT-3’, reverse primer 5’-GATGTAGGAGGGCGTGGATA-3’, 579bp product). The PCR conditions were set as follows: 1 cycle of 1 min 95°C, 30 cycles of (15 sec 95°C, 30 sec 58°C, 1 min 68°C), 1 cycle of 5 min 68°C.

To confirm recombination of the *Vhl* allele DNA was extracted from fresh frozen renal samples and genotyped by PCR using the following primers: forward primer for floxed allele 5’-CCGGAGTAGGATAAGTCAGCTGAG-3′, forward primer for recombined allele 5′-CTGGTACCCACGAAAGTGTC-3′, common reverse primer 5′-CTGACTTCCACTGATGCTTGTCACAG-3′ (400bp product for floxed allele, 200bp product for wild type and 250bp product for recombined allele). The PCR conditions used were: 1 cycle of 10 min 94°C, 55 cycles of (50 sec 95°C, 50 sec 58°C, 60 sec 72°C), 1 cycle of 5 min 72°C.

### CreER^T2^ induction by tamoxifen

Tamoxifen (Sigma, UK) was dissolved in sunflower seed oil/ethanol mixture (10:1) at 20mg/ml. Four to eight week mice were injected intraperitoneally with 100µl of tamoxifen (2mg) or sunflower seed oil per day for 5 consecutive days.

### Histology and immunohistochemistry

For detection of β-galactosidase activity, mice were euthanized either at 2 or 4 weeks post tamoxifen induction and tissues (skin, fat, pancreas, stomach, small and large intestine, spleen, liver, kidneys, bladder, genital tract, thymus, heart, lungs, muscle, salivary glands, thyroid, brain, and bone) were dissected. Samples were fixed in 10% paraformaldehyde for 1 hour, incubated for 48 hours in 20% sucrose in PBS at 4°C and then snap-frozen in liquid nitrogen and stored at -80°C. Frozen sections (5μm) were freshly cut, washed in PBS and incubated in X-gal solution (50mM Tris HCl pH 7.4, 5 mM Potassium Ferrocyanide, 5 mM Potassium Ferricyanide, 2 mM MgCl2, 0.02% NP40 and 1 mg/ml X-gal) in a humidified chamber for 18 hours at 37°C. The slides were then washed and counterstained with nuclear fast red or used for IHC.

Immunohistochemistry (IHC) was performed on either frozen or formalin fixed tissues using standard protocols. Specificity of immunostaining was assessed by incubation in the absence of primary antibody. We used the following primary antibodies: Aquaporin 1 (AQP1, NB-600-749, 1:500; Novus Biologicals), THP (AF5175, 1:100; R&D), Aquaporin 2 (AQP2, ab105171, 1:1000; Abcam), VHL (sc5575, 1:200; Santa Cruz), HIF2a (NB100-132, 1:150; Novus Biologicals), CAIX (sc-25600, 1:200; Santa Cruz) and GLUT1 (ab14683, 1:350; Abcam). Secondary antibodies used were conjugated to HRP and developed with DAB. For review of histology, slides were stained by haematoxylin and eosin (H&E). For quantification of VHL expression stained tissue sections were scanned with the Aperio ScanScope (Aperio, Vista, CA) and images were visualised and captured using the Aperio ImageScope program. Images were processed using Fiji software by calculating the percentage of DAB positive cells relative to total nuclear area of the field.

## Results

### *Pax8*-CreER^T2^ transgenic mice

#### Generation of *Pax8*-CreER^T2^ mice

**Fig 1:**
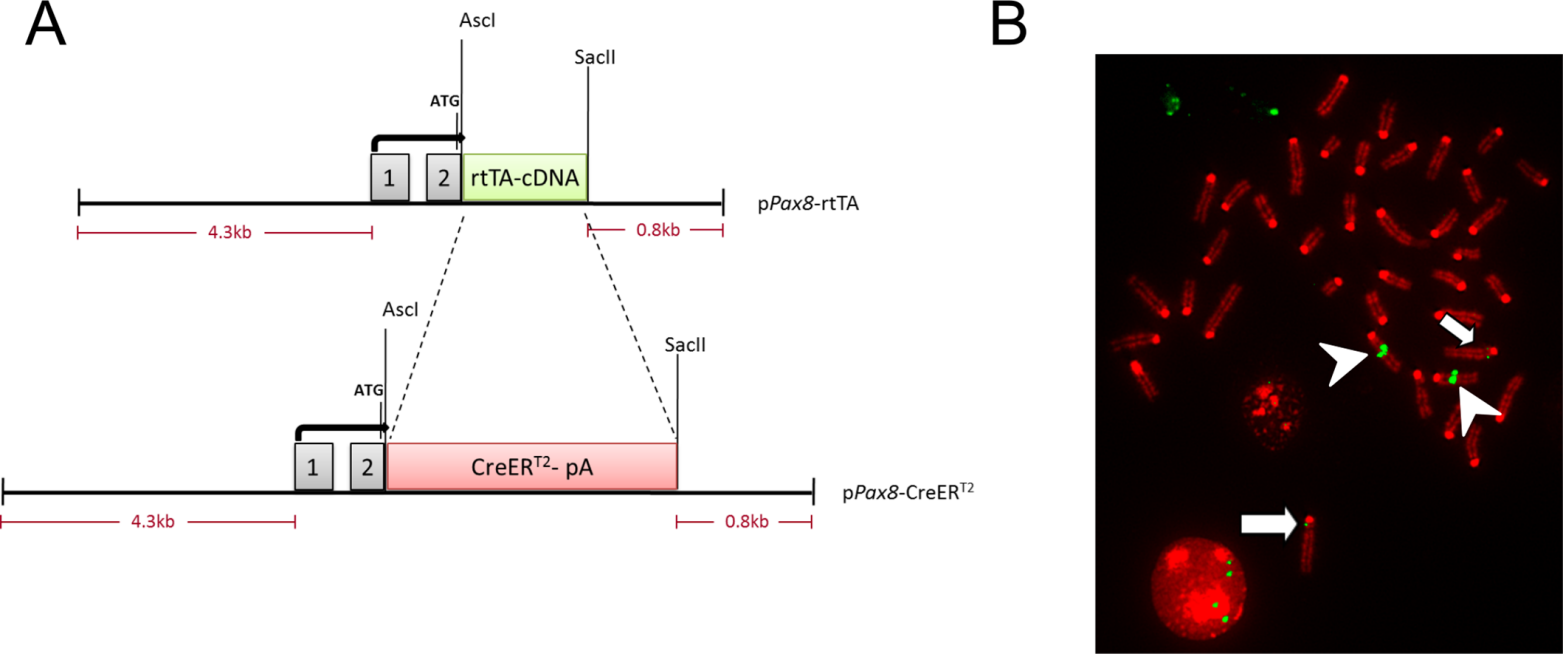
Generation of *Pax8*-CreER^T2^ transgenic mice. (A) The previously published p*Pax8*-rtTA plasmid [7] is shown in the top panel. The CreER^T2^ coding sequence followed by a polyadenylation signal (pA) was used to replace the rtTA sequence of p*Pax8*-rtTA and generate plasmid p*Pax8*-CreER^T2^ (bottom panel) containing 4.3kb of upstream regulatory sequence, complete exon 1 and intron 1, part of exon 2 and 0.8kb of intron 2 of the murine *Pax8* gene. (B) FISH analysis of metaphase spreads using p*Pax8*-CreER^T2^ as probe (green) maps the integration site to chromosome 6 (arrowheads). A weaker signal from the endogenous *Pax8* locus can be detected on chromosome 2 (arrows).

To enable targeting of all renal tubular epithelial compartments, we chose to target expression of CreER^T2^ to the kidney using the previously isolated genetic control elements of the mouse *Pax8* locus [7]. A *Pax8*-rtTA transgenic mouse model generated from this locus directs high levels of expression of the reverse tetracycline-dependent transactivator (rtTA) to all proximal, distal and collecting tubules with extrarenal activity occurring only in a minority of hepatocytes [7]. We replaced the rtTA sequence of the p*Pax8*-rtTA plasmid with a CreER^T2^ coding sequence [10] (Fig 1A). Following pronuclear injection of the 8.4-kb linearized *Pax8*-CreER^T2^ construct into C57BL/6 fertilised embryos, we identified five transgenic founders among 17 offspring mice. One of those lines displaying germline transmission was designated *Pax8*-CreER^T2^ and is described here.

Using fluorescence *in situ* hybridization we mapped the site of integration of the *Pax8*-CreER^T2^ construct to mouse chromosome 6 (Fig 1B). Paired-end whole genome sequencing was used to precisely map the transgene integration site within an intergenic region of chromosome 6qC1 (chr6:62,912,590–62,912,610; GRCh38/mm10).

#### Functional *Pax8*-CreER^T2^ activity *in-vivo*

**Fig 2:**
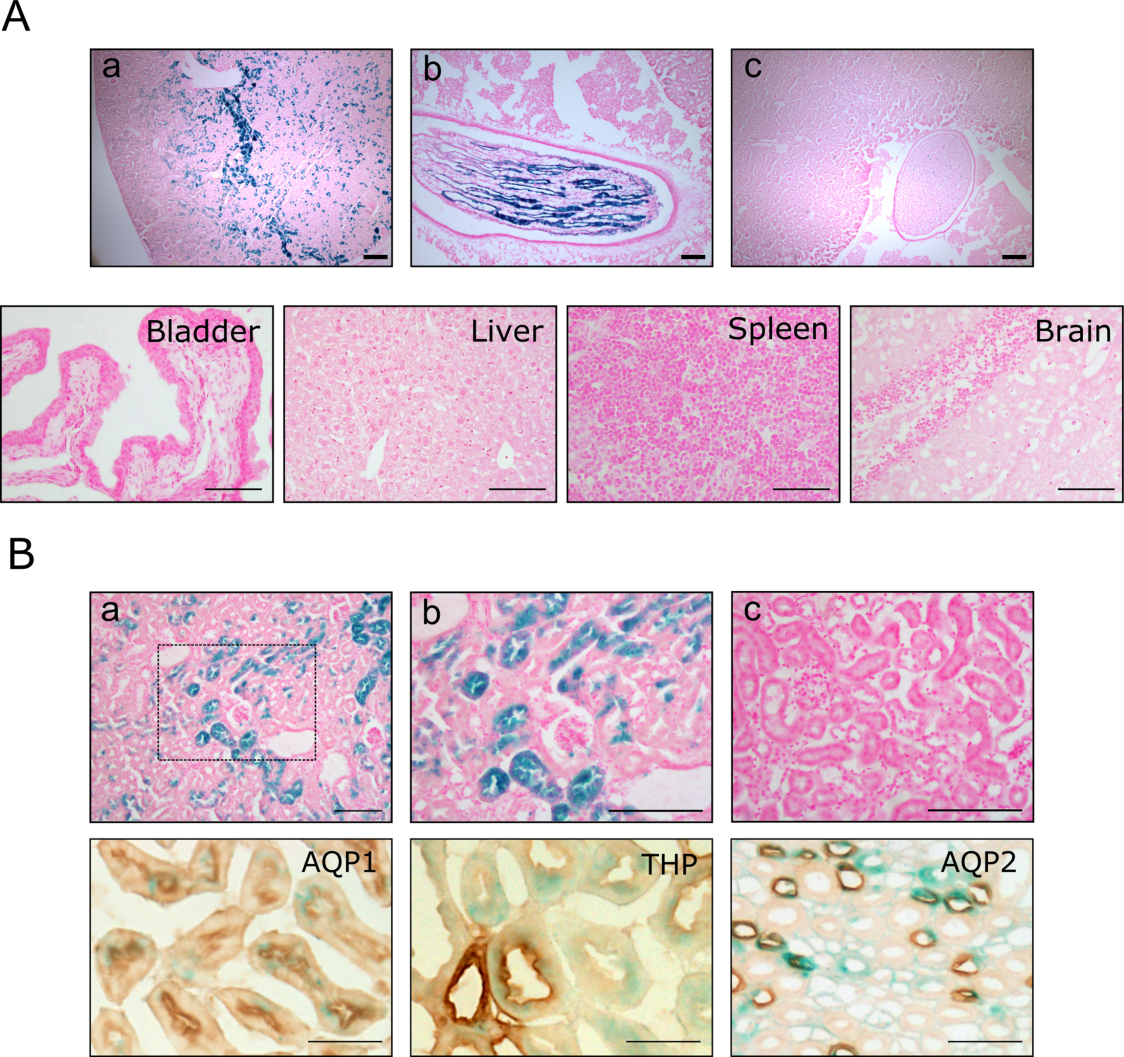
*Pax8*-CreER^T2^ mediated β-galactosidase expression. (A) Enzymatic X-gal staining of cryosections of kidneys (top panels) derived from 10 week old *Pax8*-CreER^T2^/Rosa26R mice either induced with tamoxifen (a, renal parenchyma; b, collecting ducts) or uninduced (c). Tamoxifen administration did not induce β-galactosidase expression in any of the other major organs examined (representative tissues presented in bottom panels). (B) β-galactosidase positivity was exclusively found in the tubular epithelium of the kidney of tamoxifen treated mice, with no staining observed in glomeruli or bloods vessels (a, framed area is shown enlarged in b). Kidneys from vehicle treated mice were negative (c). Co-localisation studies revealed X-gal expression within tubules of all renal tubular compartment (proximal tubules—AQP1, distal tubules–THP and collecting ducts–AQP2). Scale bars, 100μm.

To show functional expression of CreER^T2^ *in-vivo*, we crossbred *Pax8*-CreER^T2^ mice with Rosa26R reporter mice. These mice carry a lacZ gene whose expression requires excision of loxP-flanked stop sequences [15]. Double transgenic mice (*Pax8*-CreER^T2^/Rosa26R) were treated at 8 weeks of age with either tamoxifen or sunflower seed oil (vehicle control) for 5 consecutive days. Two and four weeks post treatment mice were sacrificed and kidneys and several other tissues collected. The kidneys of treated mice but not those of control showed strong β-galactosidase expression in both the cortex and medulla (Fig 2A). In contrast to the previously reported *Pax8*-rtTA transgenic mouse [7] no expression was detected in the liver of *Pax8*-CreER^T2^/Rosa26R tamoxifen treated animals. Similarly, no expression could be detected in any other tissue tested, including bladder, spleen and brain (Fig 2A).

Closer examination of the kidney revealed β-galactosidase positivity in renal tubular epithelial cells only. Parietal and visceral epithelial cells of glomeruli, mesangial cells and blood vessels did not stain (Fig 2B). Co-localisation studies with renal tubular epithelial markers revealed *Pax8*-CreER^T2^ mediated recombination in both proximal and distal tubules as well as strong β-galactosidase expression in collecting ducts (Fig 2B). Recombination specificity and efficiency did not vary between male and female mice.

### *Slc22a6*-CreER^T2^ knock-in mice

#### Generation of *Slc22a6*-CreER^T2^ mice

To enable the specific targeting of proximal renal tubular epithelia, the *Slc22a6* mouse locus was used to direct CreER^T2^ expression. *Slc22a6* encodes a protein involved in the sodium-dependent transport and excretion of organic anions (OAT1), and is exclusively expressed in the renal proximal tubules from late embryogenesis and throughout adulthood [19, 20]. As the essential elements of its promoter have not been defined, a knock-in approach was pursued for the generation of *Slc22a6*-CreER^T2^ mice.

**Fig 3:**
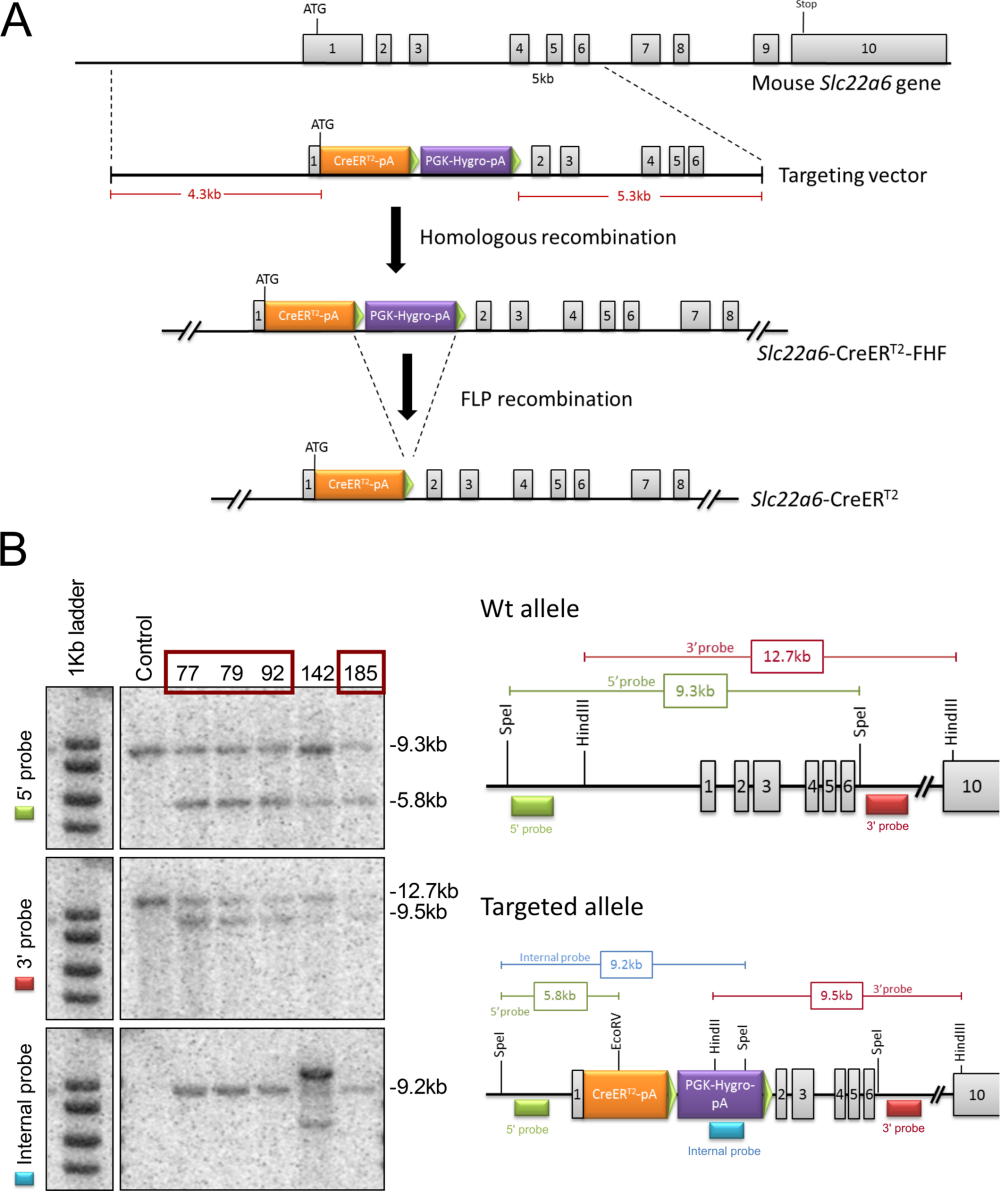
Generation of *Slc22a6*-CreER^T2^ knock-in mice. (A) Schematic diagram of the generation of Slc22a6-CreER^T2^ knock-in mice. The exon-intron structure of the mouse *Slc22a6* locus is shown at the top. The targeting vector has a 4.3 kb 5′ arm that locates immediately upstream of the *Slc22a6* gene ATG, the CreER^T2^-pA coding sequence, a PGK-hygromycin selection cassette flanked by FRT sites (green triangles) and a 5.3 kb 3′ arm that extends to intron 6. (B) Southern blots show a control (wild-type) and 4 correctly targeted ES cell clones (77, 79, 92, 185) that give expected hybridisation patterns. Clone 142 is incorrectly targeted. The location of 5’, 3’ and internal probes as well as expected band lengths are indicated in the diagrams on the right.

We constructed a targeting vector in which a CreER^T2^ cassette was inserted immediately downstream of the initiation methionine of the endogenous mouse *Slc22a6* gene, followed by a PGK-hygromycin sequence flanked by FRT sites (FHF) for selection of targeted embryonic stem (ES) cells (Fig 3A). ES cells correctly targeted with the *Slc22a6*-CreER^T2^-FHF construct were identified initially by PCR and then confirmed by Southern blotting using an external 5’ probe (9.3kb wild type and 5.8kb targeted DNA fragments from SpeI/EcoRV digested genomic DNA), an external 3’ probe (12.7kb wild-type and 9.5kb targeted DNA fragments from HindIII digested genomic DNA) and an internal probe (no wild type and 9.2kb targeted DNA fragments from SpeI digested genomic DNA) (Fig 3B). Correctly targeted ES cells were injected into C57BL/6 blastocysts to produce founder animals. Finally, the hygromycin selection cassette was removed by mating progeny to FLPeR mice [14] (confirmed using PCR analysis, data not shown) to generate *Slc22a6*-CreER^T2^ knock-in mice. Similar to conventional *Slc22a6* knockout mice [21], heterozygous and homozygous *Slc22a6*-CreER^T2^ mice were viable and displayed no discernible developmental defects (data not shown).

#### Functional *Slc22a6*-CreER^T2^ activity *in-vivo*

**Fig 4:**
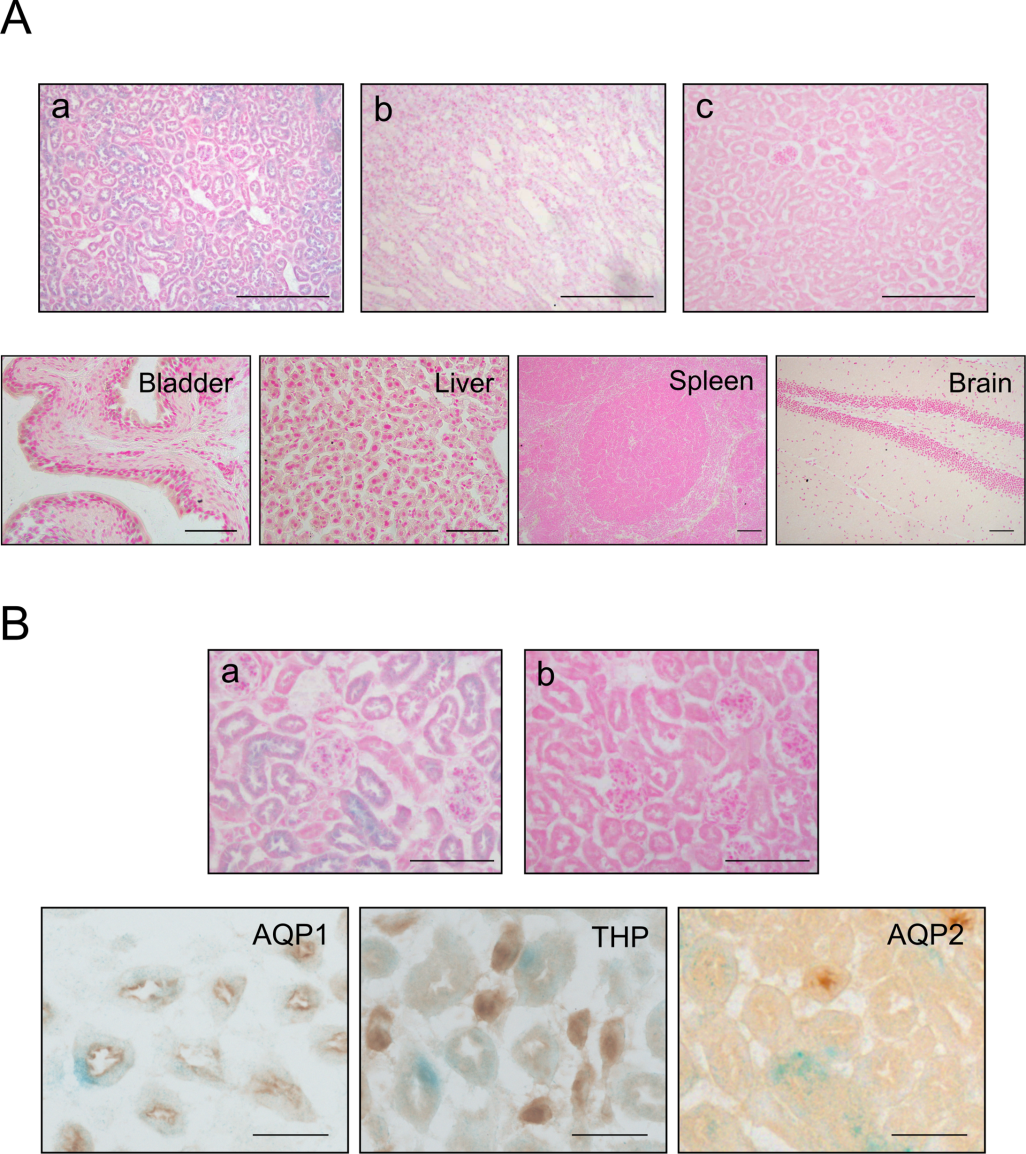
*Slc22a6*-CreER^T2^ mediated β-galactosidase expression. (A) Enzymatic X-gal staining of cryosections of kidneys (top panels) derived from 10 week old *Slc22a6*-CreER^T2^/Rosa26R mice either induced with tamoxifen (a, renal cortex; b, medulla) or uninduced (c, renal cortex). Tamoxifen administration did not induce β-galactosidase expression in any of the other major organs examined (representative tissues presented in bottom panels). (B) β-galactosidase positivity was exclusively found in the tubular epithelium of the kidney of tamoxifen treated mice (a); kidneys from vehicle treated mice were negative (b). Co-localisation studies revealed specific X-gal expression within proximal tubular epithelium (AQP1) with no expression detected in distal tubules (THP) or collecting ducts (AQP2). Scale bars, 100μm.

To show functional expression of CreER^T2^ *in-vivo*, *Slc22a6*-CreER^T2^ mice were cross-bred with Rosa26R reporter mice. Double transgenic mice (*Slc22a6*-CreER^T2^/Rosa26R) were treated at 8 weeks of age with either tamoxifen or sunflower seed oil (vehicle control) for 5 consecutive days. Two and four weeks post treatment mice were sacrificed and kidneys and several other tissues collected. The kidneys of treated mice but not those of control showed β-galactosidase expression which was limited to the renal cortex (Fig 4A). Analysis of other tissues did not reveal any expression of β-galactosidase (Fig 4A). Closer examination of the kidney revealed β-galactosidase positivity in renal tubular epithelial cells only. Parietal and visceral epithelial cells of glomeruli, mesangial cells and blood vessels did not stain (Fig 4B). Co-localisation studies with renal tubular epithelial markers revealed *Slc22a6*-CreER^T2^ mediated recombination in only proximal tubule epithelial cells and not in epithelia of distal tubules or collecting ducts (Fig 4B). Recombination specificity and efficiency did not vary between male and female mice.

### Renal tubule specific models of *Vhl* deletion

To determine the utility of our novel CreER^T2^ strains for the study of renal cancer we generated renal tubular epithelial specific deletion models of *Vhl*, a gene deleted in ~80–90% of all clear cell renal cancers [11–13]. Mice with a loxP-flanked *Vhl* allele (*Vhl*^fl/fl^) [22] were interbred with either *Pax8*-CreER^T2^ trangenic mice to induce *Vhl* deletion throughout renal tubular epithelia, or with *Slc22a6*-CreER^T2^ knock-in mice for specific proximal tubular epithelial deletion.

**Fig 5:**
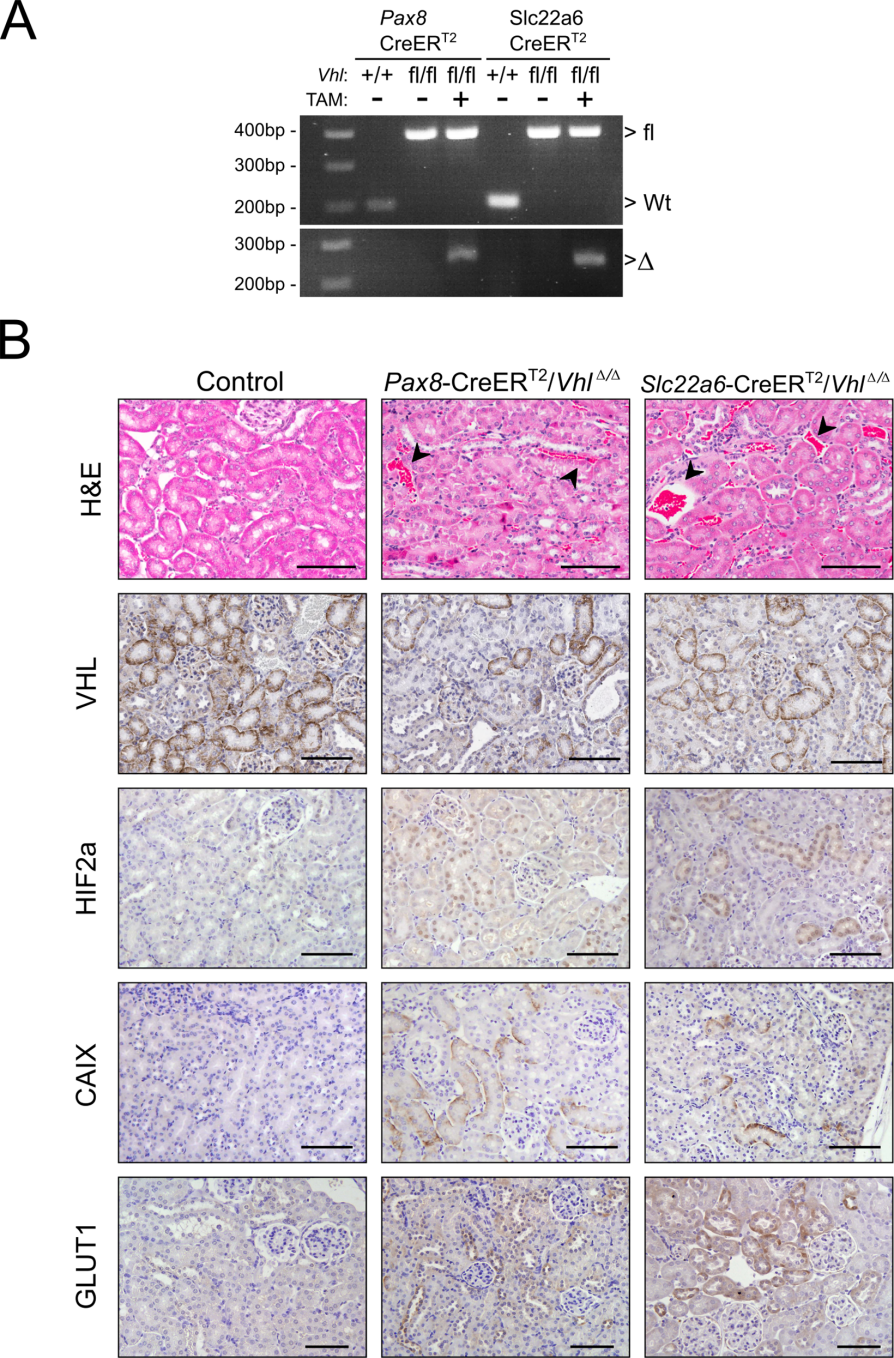
Renal tubule specific models of *Vhl* deletion. (A) PCR analysis of recombination at the *Vhl* locus in the kidneys of mice with combinations of *Pax8*-CreER^T2^, *Slc22a6*-CreER^T2^ and the *Vhl* floxed (fl) and wild-type (+) alleles. The positions of the bands representing the *Vhl* floxed, wild type (Wt) and recombined (Δ) alleles are indicated. (B) Histological images of representative renal sections from 12 month old control, *Pax8*-CreER^T2^/*Vhl*^Δ/Δ^ and *Slc22a6*-CreER^T2^/*Vhl*^Δ/Δ^ mice (stains and antibodies as indicated, arrowheads indicate abnormal vascularisation). Scale bars, 100μm.

*Pax8*-CreER^T2^/*Vhl*^fl/fl^ and *Slc22a6*-CreER^T2^/*Vhl*^fl/fl^ compound mice (n = 4 respectively) were tamoxifen induced at 4 weeks of age. Uninduced mice of the same genotype or *Vhl*^fl/fl^ mice treated with tamoxifen served as controls (n = 4). Cohorts were followed up to 15 months of age, with no evidence of any morbidity or mortality. Recombination of *Vhl* was readily detectable by genomic DNA PCR in the kidneys of tamoxifen treated compound mutant mice (hereafter referred to as *Vhl*^Δ/Δ^) but not in control animals (Fig 5A). Estimates of Vhl recombination efficiency between the two models were determined by analysis of VHL immunostaining (Fig 5B). Renal expression levels of VHL in tamoxifen treated *Pax8*-CreER^T2^/*Vhl*^Δ/Δ^ and *Slc22a6*-CreER^T2^/*Vhl*^Δ/Δ^ mice were on average 40% and 16% lower respectively than in control kidneys (S1 Fig). *Vhl*^Δ/Δ^ kidneys from both lines were of normal external appearance and parenchymal mass, and did not display histological abnormalities in the structure of the renal tubules (Fig 5B), consistent with the phenotype described in previously published human and mouse studies [23–25]. Similarly, we observed accumulation of HIF2a (a major target of the VHL protein) and its downstream targets CAIX and GLUT1, as well as an increase in cortical vascularisation [26] (Fig 5B), confirming renal specific loss of VHL in our models.

## Discussion

Here we report the generation and characterisation of two novel mouse lines, *Pax8*-CreER^T2^ and *Slc22a6*-CreER^T2^, which allow for specific and temporal modification of target genes either throughout the entire renal tubular epithelium or specifically in the proximal renal tubular compartment. We further establish their utility for the investigation of renal cancer biology by generating tractable renal tubule specific models of *Vhl* deletion.

Though a number of renal tubule targeting mouse lines have been described their utility in the investigation of renal pathophysiology and complex renal disease, like cancer, is limited by lack of inducibility [1–6, 27, 28], and cell-type and tissue specificity [7, 29–31]. In this study we set out to address this deficiency by generating novel inducible mouse lines directing genetic modifications in either specifically the proximal tubule (the presumed cell of origin of RCC [8]) or throughout all renal tubular epithelial compartments.

For renal tubule-specific modulation we chose to express the inducible CreER^T2^ cassette under the genetic control of the *Pax8* promoter. Previously described *Pax8*-rtTA transgenic mice, demonstrate highly efficient acute and chronic renal tubule targeting but are not directly compatible with the Cre-loxP recombination system, requiring interbreeding with TetO-Cre transgenic mice and hence complex breeding schedules [7]. Furthermore, kidney tubule-specific knockout of *Vhl* using the *Pax8*-rtTA line has been limited by the off-target deletion of *Vhl* in a subset of hepatocytes leading to the development of clinically significant polycythaemia [12]. The *Pax8*-CreER^T2^ transgenic line described here shows efficient and specific transgene expression in all renal tubular compartments (proximal, distal tubules and collecting ducts) with high organ specificity and no extrarenal activity detected in any of the organs analysed, including the liver. This difference in expression pattern between the *Pax8*-rtTA and *Pax8*-CreER^T2^ lines is likely to be due to the site of transgene integration (chromosome 8 band 2 and chromosome 6qC1 respectively), as exactly the same *Pax8* promoter fragment directing transgene expression is present in both lines. Importantly, the induction of *Vhl* deletion in *Pax8*-CreER^T2^/*Vhl*^fl/fl^ compound mice resulted in renal-specific loss of *Vhl* with no clinical signs of polycythaemia, thus permitting the investigation of long-term effects of *Vhl* deletion on renal physiology and disease.

Though two inducible mouse lines targeting the proximal renal tubule have been described to date (γ-glutamyl transpeptidase CreER^T2^ [30] and solute carrier family 34-member 1 CreER^T2^ [29]), their specificity of expression outside of the kidney has not been fully characterised, limiting their utility for the generation of organ-specific disease models. We thus generated and characterised a novel mouse line (*Slc22a6*-CreER^T2^) expressing a CreER^T2^ cassette from the endogenous promoter of *Slc22a6*, a gene exclusively expressed in renal proximal tubules [19, 20]. As predicted, *Slc22a6*-CreER^T2^ mice direct recombination specifically in renal proximal tubule epithelial cells with no expression detected in other renal cell types or any other extrarenal tissue examined. We further established the utility of this line for the investigation of renal cancer biology by generating *Slc22a6*-CreER^T2^/*Vhl*^fl/fl^ compound mice which phenocopied previously published models [24–26] and exhibited no limiting morbidity or mortality.

The CreER^T2^ mouse lines described in this study offer great flexibility in both the spatiotemporal distribution and the efficiency of recombination achieved. The knock-in *Slc22a6*-CreER^T2^ line drives highly specific recombination in the proximal tubules but with relatively low efficiency, as expected by integration of a single copy of the CreER^T2^ cassette in a native mouse locus. This line represents a powerful tool for the study of clonal physiology and pathology of the proximal tubule, such as in studies of cell fate determination during development, tissue maintenance and repair (lineage tracing and genetic labelling approaches) and the investigation of renal cancer initiation and progression. In contrast, the *Pax8*-CreER^T2^ transgenic line (multiple copy integration) allows for the genetic modification of multiple renal tubular subtypes with high efficiency permitting more general, agnostic approaches in the investigation of renal biology and disease.

In this study we restricted analyses to CreER^T2^ induction of fully developed renal tubular epithelia. The *Pax8*-CreER^T2^ and *Slc22a6*-CreER^T2^ mouse lines would also permit targeting of renal tubules within early stages of development. Determination of the optimum dosing schedule and efficiency of recombination at embryonic or neonatal tissues will require investigation in additional studies.

Modelling clinically relevant renal disease requires the accurate genetic modification of relevant cell types (cell of origin) within an appropriate time frame (early organogenesis versus full differentiation). The *Pax8*-CreER^T2^ and *Slc22a6*-CreER^T2^ mouse lines described here exhibit high tissue specificity and inducibility and therefore represent powerful new tools for renal research.

## Supporting Information

S1 Fig *Pax8*-CreER^T2^/*Vhl*^Δ/Δ^ and *Slc22a6*-CreER^T2^/*Vhl*^Δ/Δ^ recombination efficiency. Representative images of VHL immunohistochemistry (A) and corresponding relative quantification of VHL protein expression levels (B) in the kidneys of 12 month old control (n = 3), *Pax8*-CreER^T2^/*Vhl*^Δ/Δ^ (n = 4) and *Slc22a6*-CreER^T2^/*Vhl*^Δ/Δ^ (n = 4) mice. Data represent mean ± s.e.m.
(TIF)
