# Supplementary figures and images for: Generation and Characterisation of a Pax8-CreERT2 Transgenic Line and a Slc22a6-CreERT2 Knock-In Line for Inducible and Specific Genetic Manipulation of Renal Tubular Epithelial Cells
Source: PLoS One. 2016 Feb 11;11(2):e0148055. doi: 10.1371/journal.pone.0148055 (PMC4751286; doi:10.1371/journal.pone.0148055)

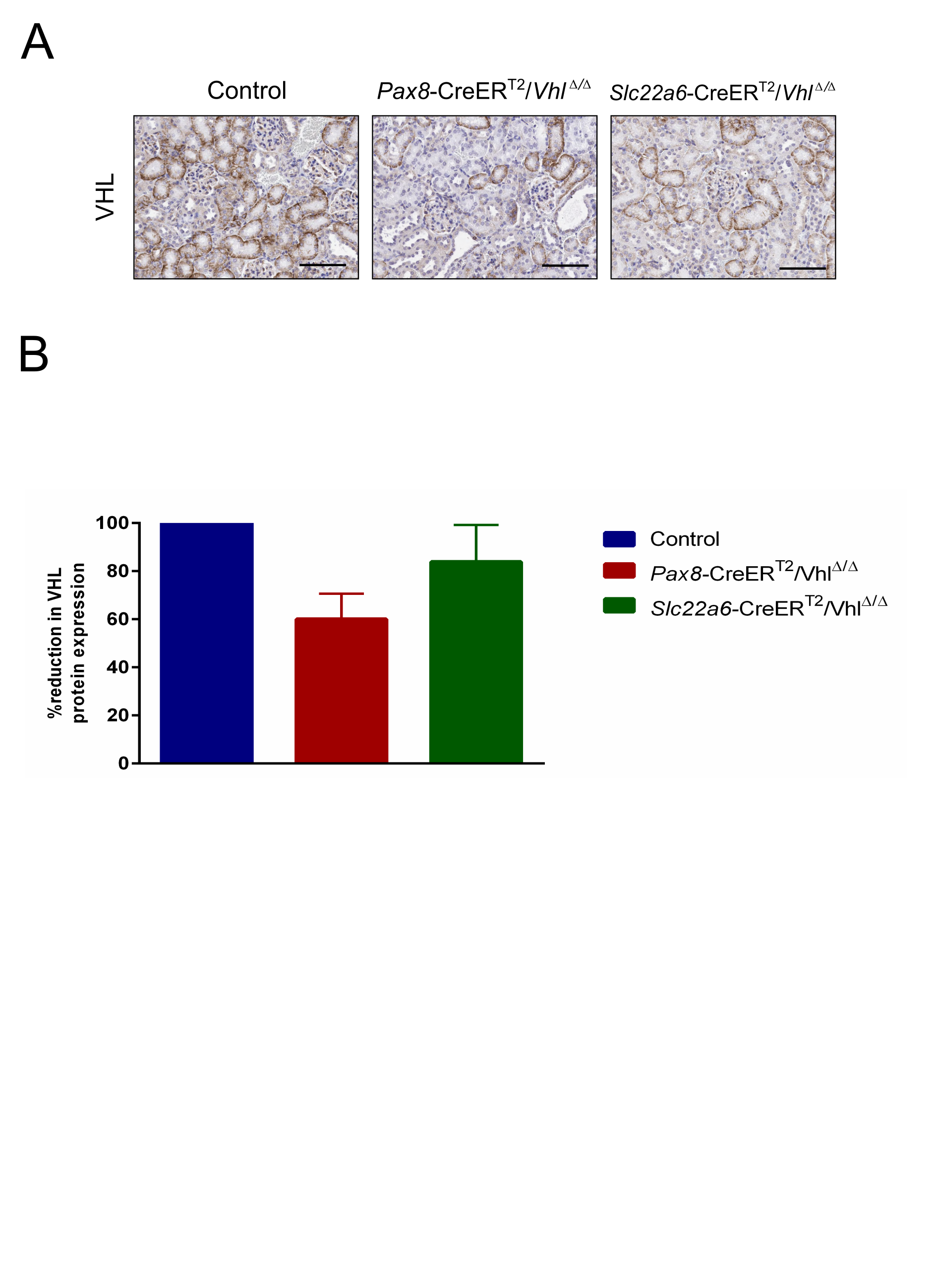

Supplement: S1 Fig — Representative images of VHL immunohistochemistry (A) and corresponding relative quantification of VHL protein expression levels (B) in the kidneys of 12 month old control (n = 3), Pax8-CreERT2/VhlΔ/Δ (n = 4) and Slc22a6-CreERT2/VhlΔ/Δ (n = 4) mice. Data represent mean ± s.e.m. (TIF) [file pone.0148055.s001.tif]
